# Extracellular Vesicles from BOEC in *In Vitro* Embryo Development and Quality

**DOI:** 10.1371/journal.pone.0148083

**Published:** 2016-02-04

**Authors:** Ricaurte Lopera-Vásquez, Meriem Hamdi, Beatriz Fernandez-Fuertes, Verónica Maillo, Paula Beltrán-Breña, Alexandra Calle, Alberto Redruello, Soraya López-Martín, Alfonso Gutierrez-Adán, María Yañez-Mó, Miguel Ángel Ramirez, Dimitrios Rizos

**Affiliations:** 1 Departamento de Reproducción Animal, Instituto Nacional de Investigación y Tecnología Agraria y Alimentaria, INIA, Madrid, Spain; 2 Hospital Universitario Santa Cristina, Instituto de Investigaciones Sanitarias Princesa (IIs-IP), Madrid, Spain; 3 Departamento de Biología Molecular, UAM/CBM-SO, Madrid, Spain; Hull York Medical School, UNITED KINGDOM

## Abstract

To evaluate the effect of conditioned media (CM) and Extracellular Vesicles (EVs) derived from bovine oviduct epithelial cell (BOEC) lines on the developmental capacity of bovine zygotes and the quality of embryos produced *in vitro*, presumptive zygotes were cultured under specific conditions. In experiment 1, zygotes were cultured either on monolayers from BOEC extended culture (E), together with fresh BOEC suspension cells, or with BOEC-CM from fresh or E-monolayers. In experiment 2, EVs were isolated from BOEC-CM and characterized (150–200 nm) by Nanosight^®^ and electron microscopy. Zygotes were cultured in the presence of 3x10^5^EVs/mL, 1.5x10^5^EVs/mL or 7.5x10^4^EVs/mL of fresh or frozen BOEC-EVs. In experiment 3, zygotes were cultured in absence of FCS but with EVs from BOEC-E that had been cultured in different culture media. In experiment 4, zygotes were cultured in SOF+5% normal-FCS, or EV-depleted-FCS. In all cases, cleavage rate (Day 2) and blastocyst development (Day 7–9) was assessed. Blastocysts on Days 7/8 were used for quality evaluation through differential cell count, cryotolerance and gene expression patterns. No differences were found among all FCS-containing groups in cleavage rate or blastocyst yield. However, embryos derived from BOEC-CM had more trophectoderm cells, while embryos derived from BOEC-EVs, both fresh and frozen, has more trophectoderm and total cells. More embryos survived vitrification in the BOEC-CM and BOEC-EV groups. In contrast, more embryos survived in the EV-depleted-FCS than in normal-FCS group. Gene expression patterns were modified for *PAG1* for embryos cultured with EVs in the presence of FCS and for *IFN-T*, *PLAC8*, *PAG1*, *CX43*, and *GAPDH* in the absence of FCS. In conclusion, EVs from FCS have a deleterious effect on embryo quality. BOEC-CM and EVs during *in vitro* culture had a positive effect on the quality of *in vitro* produced bovine embryos, suggesting that EVs have functional communication between the oviduct and the embryo in the early stages of development.

## Introduction

*In vitro* embryo production is a useful tool to study early embryonic development in mammals, to solve reproductive issues in humans and to conserve gametes from animals with high genetic merit or endangered species. However, despite scientific efforts to improve the performance of *in vitro* production systems, the quality of such embryos remains lower than those produced *in vivo*, resulting in increased embryo losses.

*In vitro* conditions are suboptimal, as evidenced by lower blastocyst yields (30–40%), lower cryotolerance [1], altered inner cell mass/trophectoderm cells ratio [2], altered gene expression patterns [3], and lower pregnancy rates of transferable embryos [4].

The first stages of bovine embryo development occur in the oviduct, where the embryo spends around 4 days [5]. The oviduct is an active organ that maintains and modulates the milieu for sperm capacitation, transport and fertilization of the mature oocyte and early embryonic development [6–8]. The embryo in the oviduct undergoes epigenetic changes responsible for further development, implantation and postnatal phenotype [9]. At a molecular level, Embryonic Genome Activation (EGA), the time at which the embryo starts to synthetize and use its own mRNA, is the most important step and occurs at the 8–16 cell stage [10], ensuring normal preimplantation and early fetal development [11].

The oviductal environment can support embryonic growth up to the blastocyst stage across a wide range of species after trans-species transfer [12]. For example, the ligated sheep oviduct can provide an adequate environment, not only for sheep embryos but also for those from other farm species, including cattle [13]. Culture of *in vitro* produced zygotes in the ewe oviduct did not affect blastocyst yields but clearly improved the quality of the blastocysts, as measured by survival after cryopreservation [14] and pregnancy rates [15].

The exchange of signals between the embryo and the oviduct is remarkable, although the molecular mechanisms involved in this embryo-maternal communication are currently mostly unknown [16]. The epithelium of the oviduct is made up of ciliary and secretory cells which secrete proteins and other factors that contribute to the development of the early embryo [17]. Bovine oviduct epithelial cells (BOEC) take part in intimate contact with gametes and embryos during fertilization and early embryo development, and are considered the most suitable *in vitro* model to study early embryonic maternal interactions [18,19], with positive outcomes when included in embryo production systems [20]. BOEC modify their transcription in the presence of developing embryos [21], demonstrating that co-culture systems allow a dynamic exchange of nutrients and cell secretions [22]. BOEC secrete growth and embryotrophic factors into the culture media [23] and change the culture metabolites [24] required by the embryos. Thus, BOEC have been shown to help overcome the developmental block occurring at the 8- to 16-cell stage in *in vitro* produced cattle embryos [25].

However, co-culture is associated with methodological complexity, lack of reproducibility and biosanitary risk [26]. One alternative to reduce the variability in such systems could be the use of cell lines that maintain primary culture attributes [19]. An even better alternative may come from conditioned media (CM) culture systems, which pose several advantages over the co-culture, such as the absence of foreign cells and the presence of embryotrophic factors [27] that support the development of early bovine embryos [28], giving insights into the mechanism(s) by which epithelial cells support the development of embryos [29].

Recent studies have demonstrated that membrane-enclosed vesicles, collectively named Extracellular Vesicles (EVs), released by somatic cells, contain bioactive molecules (i.e., proteins and RNAs, mRNAs, miRNAs [30] and lipids [31]), and are present in some bodily fluids [32]. It has been demonstrated that EVs can horizontally transfer functional RNAs to other cells [33–35]. Thus, EVs are an important tool in intercellular communication playing a key role in the regulation of several physiological and pathological processes [36]. In reproduction, secreted vesicles are present in the follicular fluid [37], endometrial environment [38] and seminal plasma [39].

Given this background, the aim of the present study was to produce an *in vitro* system better resembling the pre-implantation embryo environment, using a culture system incorporating EVs obtained from BOEC, for improving embryo development and quality.

## Materials and Methods

Unless otherwise stated, all chemicals were purchased from Sigma Aldrich Química S.A Company (Madrid, Spain).

### Oocyte collection and *in vitro* maturation

Immature cumulus oocyte complexes (COCs) were obtained by aspirating follicles (2-8mm) from the ovaries of mature heifers and cows collected at slaughter from a local abattoir (Transformación Ganadera De Leganés S.A., Madrid, Spain). Class 1 and 2 COCs (homogenous cytoplasm and intact cumulus cells) were matured for 24 h in 500μL of maturation media (TCM-199 supplemented with 10% (v/v) foetal calf serum (FCS), and 10 ng/ml epidermal growth factor) in four well dishes, in groups of 50 COCs per well at 38.5°C under an atmosphere of 5% CO_2_ in air, with maximum humidity.

### Sperm preparation and *in vitro* fertilization

Frozen semen from an Asturian Valley bull (ASEAVA, Asturias, Spain), was thawed at 37°C in water bath for 1 minute and centrifuged for 10 minutes at 280 x*g* through a gradient of 1 ml of 40% and 1 ml of 80% Bovipure^®^ according to the manufacturer´s specification (Nidacon Laboratories AB, Göthenborg, Sweden). The sperm pellet was isolated and washed in 3 ml of Boviwash^®^ (Nidacon) by centrifugation at 280 x*g* for 5 min. The pellet was re-suspended in the remaining 300 μl of Boviwash^®^. Sperm concentration was determined and adjusted at a final concentration of 1x10^6^ sperm/ml for the IVF. Gametes were co-incubated for 18–22 h in 500μL of fertilization media (Tyrode's medium with 25 mM bicarbonate, 22 mM Na lactate, 1mM Na-pyruvate, and 6 mg/ml fatty acid-free BSA supplemented with 10 mg/ml heparin sodium salt, Calbiochem, San Diego, CA) in a four well dish, in groups of 50 COCs per well under an atmosphere of 5% CO_2_ in air, with maximum humidity at 38.5°C.

### *In vitro* culture of presumptive zygotes

**Fig 1:**
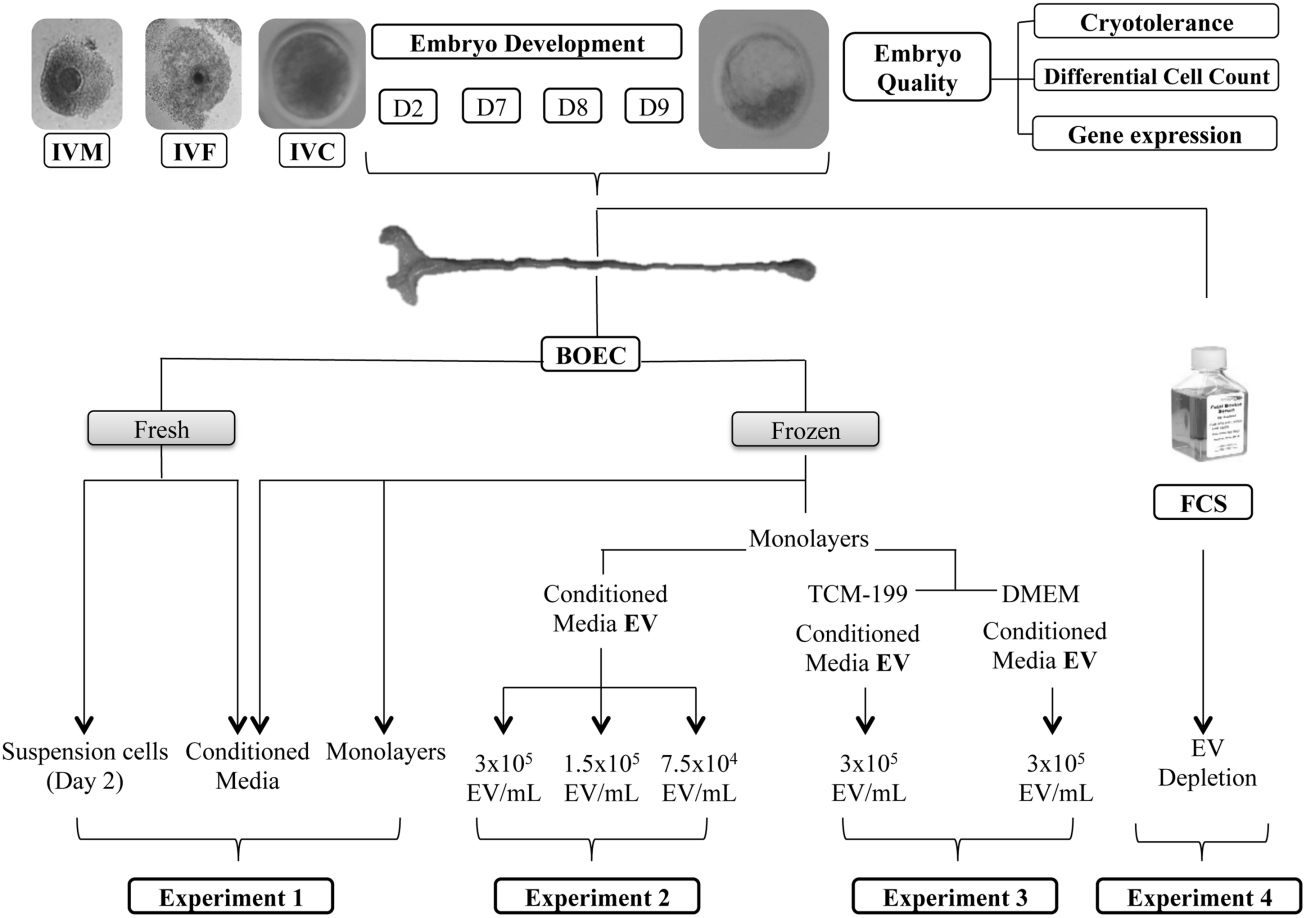
Experimental design.

At approximately 20 h post-insemination (p.i.), presumptive zygotes were denuded of cumulus cells by vortex and cultured in groups of 25 in 25 μl droplets of Synthetic Oviduct Fluid, (SOF) [40]—with 4.2 mM sodium lactate, 0.73 mM sodium pyruvate, 30 μl/ml BME amino acids, 10 μl/ml MEM amino acids, 1 μg/ml phenol-red with and without BOEC or in CM or cultured with EVs (see experimental design for clarification) under mineral oil at 38.5°C under an atmosphere of 5% CO_2_, 5% O_2_ and 90% N_2_. For certain experiments (Fig 1), SOF was supplemented with 5% FCS. Half of the media in BOEC embryo co-culture drops was replaced every 48 h.

### Assessment of embryo development and quality

#### Embryo development

Cleavage rate was recorded at Day 2 (48 h post insemination) and cumulative blastocyst yield was recorded at Days 7, 8, and 9 p.i. under a stereomicroscope.

#### Blastocyst vitrification

The ability of the blastocyst to withstand cryopreservation was used as quality indicator. Day 7 and 8 blastocysts were vitrified in holding medium (HM) (TCM199-M7528 supplemented with 20% (v/v) FCS) and cryoprotectants, following the procedures of Rizos *et al*. [14], in a two step protocol using the Cryoloop^®^ device (Hampton Research. Aliso Viejo, CA). First step: HM with 7.5% ethylene glycol, 7.5% dimethyl sulfoxide. Second step: HM with 16.5% ethylene glycol, 16.5% dimethyl sulfoxide and 0.5M Sucrose. The blastocysts were then warmed in two steps in HM with 0.25 M and 0.15 M sucrose and then cultured in 25 μl droplets of SOF with 5% FCS. Survival was defined as re-expansion of the blastocoel and its maintenance for 24, 48, and 72 h.

#### Differential staining of blastocysts

Differential staining of inner cell mass (ICM) and trophectoderm (TE) cells was carried following the procedures of Thouas *et al*. [41]. Briefly, blastocysts were permeabilized and TE cells were stained by incubation in 500 μl PBS with 0.2% Triton X-100 and 100 μg/ml propidium iodide (PI) in the dark for 60 sec at 37°C. For fixation and ICM staining, blastocysts were transferred into 500 μl pure ethanol with 25 μg/ml bisbenzimide (Hoechst 33342) for 3 min. Fixed and stained blastocysts were transferred to glycerol and mounted onto a glass microscope slide, gently flattened with a coverslip and visualized for cell counting under a fluorescent microscope.

#### Gene expression analysis

Poly (A) RNA was extracted from three groups of pools of 10 blastocysts from each experimental group using the Dynabeads mRNA Direct Extraction Kit (Dynal Biotech. Oslo, Norway) with minor modifications [42]. Immediately after extraction, reverse transcription (RT) was performed in accordance with manufacturer’s instructions (Bioline, Ecogen. Madrid, Spain) using poly(T) primer, random primers, and MMLV reverse transcriptase. Quantification of cDNA was realized using SYBR Green II (Molecular Probe 07568) by running the "DNA concentration measurement" module on a qPCR machine (Rotor Gene 3000, Corbett Research, Australia). The quantification of all mRNA transcripts was carried out by qPCR with two repetitions for all genes of interest. qPCR was performed by adding a 2 μl aliquot of each cDNA sample (60 ng/μl) to the PCR mix containing the specific primers. Primer sequences and the approximate sizes of the amplified fragments of all transcripts are shown in Table 1. For quantification, qPCR was performed as described previously [43]; PCR conditions were tested to achieve efficiencies close to 1. The comparative cycle threshold (CT) method was used to quantify expression levels. Values were normalized to the endogenous control (housekeeping (HK) genes: histone *H2AFZ* and 18 s ribosomal RNA (*18S*)). Fluorescence was acquired in each cycle to determine the threshold cycle or the cycle during the log-linear phase of the reaction at which fluorescence increased above background for each sample. According to the comparative CT method, the ΔCT value was determined by subtracting the HK mean CT value for each sample from each gene CT value of the sample. The calculation of ΔΔCT involved using the highest treatment ΔCT value, i.e. the treatment with the lowest target expression, as an arbitrary constant to subtract from all other ΔCT sample values. Fold changes in the relative gene expression of the target were determined using the formula 2^-ΔΔCT^ [44].

**Table 1:** Primers used for RT-qPCR.

Gene Name	Primer Sequence (5´- 3´)		Fragment size (bp)	GenBank access No.
*PLIN2*	Forward	ACAACACACCCCTCAACTGG	211	NM_173980.2
	Reverse	CTGCCTGCCTACTTCAGACC		
*ACACA*	Forward	AAGCAATGGATGAACCTTCTTC	196	FN185963.1
	Reverse	GATGCCCAAGTCAGAGAGC		
*IFN-τ*	Forward	GCCCTGGTGCTGGTCAGCTA	564	AF238612
	Reverse	CATCTTAGTCAGCGAGAGTC		
*PLAC8*	Forward	CGGTGTTCCAGAGGTTTTTCC	163	NM_016619
	Reverse	AAGATGCCAGTCTGCCAGTCA		
*PAG1*	Forward	CAACGTGCCATTTCTGAGCCTG	115	NM_174411.2
	Reverse	TGATGTCCCGGTGTCCACAAGG		
*DNMT3A*	Forward	CTGGTGCTGAAGGACTTGGGC	318	XM_001252215.1
	Reverse	CAGAAGAAGGGGCGGTCATC		
*TFAM*	Forward	GGCAGACTGGCAGGTGTA	164	AF311909
	Reverse	CGAGGTCTTTTTGGTTTTCCA		
*CX43*	Forward	TGGAATGCAAGAGAGGTTGAAAGAGG	293	NM_174068.2
	Reverse	AACACTCTCCAGAACACATGATCG		
*GPX1*	Forward	GCAACCAGTTTGGGCATCA	116	NM_174076.3
	Reverse	CTCGCACTTTTCGAAGAGCATA		
*MnSOD*	Forward	CCCATGAAGCCTTTCTAATCCTG	307	S67818.1
	Reverse	TTCAGAGGCGCTACTATTTCCTTC		
*GLUT1*	Forward	AGCGTCATCTTCATCCCAGC	540	NM_174602.2
	Reverse	CCACAATGCTCAGGTAGGAC		
*GAPDH*	Forward	GGGCGTGAACCACGAGAAGTA	120	NM_001034034.2
	Reverse	CCCTCCACGATGCCAAAGT		
*G6PD*	Forward	CGCTGGGACGGGGTGCCCTTCATC	347	NM_001244135.1
	Reverse	CGCCAGGCCTCCCGCAGTTCATCA		
*H2AFZ*	Forward	AGGACGACTAGCCATGGACGTGTG	208	NM_174809
	Reverse	CCACCACCAGCAATTGTAGCCTTG		
*18S*	Forward	AGAAACGGCTACCACATCCAA	45	NR_036642.1
	Reverse	CCTGTATTGTTATTTTTCGTCACTACCT		

### Bovine oviduct epithelial cells (BOEC)

Oviducts ipsilateral to the corpus luteum at the mid-luteal phase of the estrous cycle were collected from heifers at local slaughterhouse, sealed in a plastic bag and transported to the laboratory on ice. Each oviduct was trimmed free of tissue and oviductal mucosa was collected by squeezing and washed 2 times with PBS by centrifugation at 300 x g for 10 min. The pellet was resuspended in 2 ml of trypsin-EDTA and incubated for 3 min at 37°C. The action of the trypsin was blocked with 2 ml of SOF + 5% of FCS, pipetting until obtaining a single cell suspension. BOEC were counted in a hemocytometer, diluted to a final concentration of 1x10^6^ cells/ml and plated for culture at 38.5°C, 5% CO_2_ and saturated humidity until confluence. Half of the media (SOF+5% FCS) were replaced every 48 h.

BOEC were cultured in 4-well dishes with SOF+5% FCS for suspension cells and conditioned media production.

For BOEC extended culture (BOEC-E), cells were cultured in 100 mm petri dish with Dulbecco’s modified Eagle medium (DMEM plus 4.5 mg/L glucose, GlutaMAX, and pyruvate; Invitrogen, Carlsbad, CA) supplemented with 10% FCS, 2mM glutamine, 1mM MEM nonessential amino acids solution, and antibiotics (100 U/ml penicillin, 100 mg/ml streptomycin). At Day 5 to 7 of BOEC culture (DMEM), when cell confluence was 100%, monolayers were frozen in FCS+10% DMSO at -80°C. After thawing, cells were cultured until 100% confluence and used for embryo co-culture or CM production as explained above. The same BOEC-E frozen/thawed line was used for all experiments. Analysis of the expression of epithelial markers (Cadherin and Cytokeratin) revealed no contamination of stromal cells. A faint vimentin upregulation could be detected after a few passages, as expected for extended cell cultures (S1 Fig)

#### Suspension cells preparation

At Day 2 of cell culture (SOF+5% FCS), BOEC suspension cells were isolated from the supernatant media and washed twice before used for embryo culture.

### Inmunofluorescence stainings

BOEC-E cells were grown to confluence on glass coverslips coated with 5% gelatin and fixed with 4% paraformaldehyde (Panreac) for 1 h. Samples were permeabilized for 5min with 0.5% Tx-100, washed and stained with the appropriate primary and secondary antibodies (anti-bovine-pancadherin, anti-bovine-pancytokeratin and anti-bovine-vimentin, all from Sigma). Then samples were mounted with DAPI-containing Prolong (Invitrogen) and visualized under an epifluorescence microscope.

### Conditioned media (CM) preparation

At Day 5 to 7 of BOEC culture (SOF+5% FCS), when cell confluence was 100%, monolayers were washed with PBS before new SOF+5% FCS was added for CM production after additional 72 h of culture. Then supernatant was filtered through a 0.22 μm nitrocellulose membrane and used for embryo culture or EVs isolation.

### Extracellular vesicles isolation and quantification

EVs were isolated from BOEC-ECM, by ultracentrifugation following the procedures of Théry *et al*. [45]. Briefly, filtered BOEC-ECM was centrifuged at 100000 x*g* for 60 min at 4°C (Avanti J30i, Beckman Coulter). Then, the supernatant was removed and the pellet was re-suspended in PBS for EVs washing by repeating the previous step. An aliquot (100 μl) of the resultant pellet (resuspended in 400 μl) was used to determine the size and number of EVs by Nanoparticle Tracking Analysis (NTA) with Nanosight^®^ LM10 and NTA 2.3 Software (Nanosight, Wiltshire, UK), and transmission electron microscopy. After quantification, the EVs concentration was standardized and either frozen or used fresh for embryo culture.

### Extracellular vesicles-depleted FCS

Heat inactivated FCS (F2442) was subjected to overnight (18 h) centrifugation at 100000 x*g* at 4°C (Avanti J30i, Beckman Coulter). Then the supernatant were aliquoted and stored at -20°C for embryo culture media supplement.

### Transmission electron microscopy

For negative staining of EVs, ionized carbon and collodion-coated copper electron microscopy grids were floated on a sample drop, washed, and stained with 2% uranyl acetate (in double-distilled water) for 1 min and visualized in a JEM-1010 (JEOL, Tokyo, Japan) transmission electron microscope.

### Bead-assisted flow cytometry of extracellular vesicles

EV preparations were coupled overnight to aldehyde/sulphate-latex beads (4 μm; Invitrogen, Carlsbad, CA), and stained with primary (anti-CD9 mAb VJ1/20 [46] anti-bovine CD63 (Serotec), anti-ERM 90:3 pAb [46] and anti-TSG101 mab (Abcam)) followed by appropriate secondary antibodies. For TSG101 and ERM staining, primary and secondary antibodies were diluted in Facs Lysing solution (BD). All other samples antibodies were diluted in wash buffer (PBS supplemented with 0.1% BSA and 0.01% NaN3). Samples were analysed by standard flow cytometry in a Cytomics FC 500 MPL cytometer (Beckman Coulter).

### Western-blot

EV preparations were lysed in non-reducing Laemmli loading buffer and resolved in a 4–25% gradient SDS-PAGE gel (Biorad). Proteins were transferred to a PVDF membrane (Biorad), blocked with 10% skimmed milk and incubated with the following primary antibodies: anti-CD9 mAb VJ1/20 [46], anti-ERM 90:3 pAb [46] and anti-TSG101 mab (Abcam)) followed by peroxidase-coupled secondary antibodies and revealed detected by chemiluminescence with an ImageQuant LAS4000 biomolecular imager (GE LifeSciences).

### Experimental design (see Fig 1)

#### Experiment 1: Effect on embryo development and quality of *in vitro* culture with different types of BOEC and conditioned media

The developmental capacity of bovine zygotes and the quality of the produced embryos were assessed on Day 7–8 of *in vitro* culture under the following conditions: SOF+5% FCS, Control group (used as a basic medium for the remaining groups—C^+^); BOEC suspension cells (BOEC-S); BOEC extended culture monolayer (BOEC-E); CM from fresh BOEC monolayer (BOEC-CM); and CM from BOEC-E monolayer (BOEC-ECM).

BOEC primary cultures were prepared 3–7 days before embryo culture starts. At approximately 20 h p.i., presumptive zygotes were transferred to droplets for embryo culture according to the experimental groups. Half of the media was replaced every 48 h. Overall cleavage rate was recorded at 48 h pi and blastocyst development was recorded on Days 7, 8, and 9 p.i. A representative number of Day 7–8 blastocysts from each group were either vitrified/warmed for survival rate analysis every 24 h up to 72 h post-warming, fixed for differential cell count, or frozen in Liquid N_2_ in groups of 10 and stored at -80°C for gene expression analysis. A total of 11 replicates were carried out.

#### Experiment 2: Effect of extracellular vesicles from BOEC on the development and quality of *in vitro* produced bovine embryos

The developmental capacity and quality of bovine zygotes cultured in the presence of EVs previously isolated from BOEC-E conditioned media were assessed. At approximately 20 h post insemination, presumptive zygotes were transferred to culture droplets for embryo culture with recently isolated EVs (“fresh” EV) or frozen/thawed EVs (Fr-EV) diluted in SOF+5% FCS (C^+^) at different concentrations: 3x10^5^ EV/ml; 1.5x10^5^ EV/ml; and 7.5x10^4^ EV/ml. Because of the lack of information in the literature of EVs physiological concentrations, we took as starting dilution the initial concentration of secreted vesicles (3x10^5^ EV/ml = 100%) recovered from 10 mL of CM produced from a confluent BOEC-E monolayer in a 100 mm petri dish (≈5.5x10^6^ cells). After isolation and characterization, BOEC EVs were diluted and either frozen/thawed or used fresh for embryo culture. Blastocyst development and quality was assessed as in Experiment 1. A total of 13 replicates were carried out.

#### Experiment 3: Effect of extracellular vesicles secreted from BOEC cultured in different culture media (DMEM or TCM199) on the development and quality of *in vitro* produced bovine embryos in the absence of FCS

In this experiment the developmental capacity and quality of bovine zygotes cultured *in vitro* in the absence of FCS, in the presence or not of previously isolated EVs from BOEC-E conditioned media, that had been cultured with specific cell media, were assessed. At approximately 20 h post insemination presumptive zygotes were cultured with frozen EVs (from CM of BOEC-E cultured either in DMEM or in TCM199) in the absence of FCS in SOF (C^-^) with 3x10^5^ EV/ml. A positive control of SOF+5% FCS (C^+^) was included as well. Blastocyst development and quality was assessed as mentioned in Experiment 1. A total of 8 replicates were carried out.

#### Experiment 4: Effect of extracellular vesicles present in FCS on *in vitro* bovine embryo development and embryo quality

In this experiment the developmental capacity and quality of bovine zygotes cultured *in vitro* with normal FCS or EVs-depleted FCS were assessed. At approximately 20 h post insemination presumptive zygotes were cultured in SOF+5% FCS, containing EV (+) or EV-depleted (-). Embryo development and survival after vitrification/warming was assessed. A total of 4 replicates were carried out.

### Statistical analysis

Data on cleavage rates, blastocyst yield, survival after vitrification/warming and relative mRNA abundance for candidate genes were analyzed using one-way analysis of variance ANOVA (p<0.05). The embryo cell number (ICM, TE and Ratio) was analyzed by multiple pair-wise comparisons using a t—test. All analyses were made with the SigmaStat (Jandel Scientific, San Rafael, CA) software package.

## Results

### The use of conditioned media from a BOEC extended culture monolayer has a positive effect on the quality of bovine embryos

We analyzed embryos cultured either in SOF+5% FCS (Control group); in coculture with BOEC suspension cells (BOEC-S) or a monolayer of BOEC extended cultures (BOEC-E) or in the presence of CM from monolayers of fresh (BOEC-CM) or BOEC-E (BOEC-ECM). No differences were found in terms of cleavage rates (range: 87.6–89.4%) or blastocyst yield on Day 7 (range: 21.7–27.7%), Day 8 (range: 31.0–36.0%) or Day 9 (range: 34.3–39.3%) between groups, as shown in Table 2.

**Table 2:** Effect of co-culture with different types of BOEC and Conditioned Media on embryo development in vitro.

			Blastocyst yield		
	n	Cleavage n (% ± S.E)	Day 7 n (% ± S.E)	Day 8 n (% ± S.E)	Day 9 n (% ± S.E)
Control (C^+^)	682	599 (87.8±1.2)	176 (26.0±2.4)	217 (32.0±2.3)	237 (35.2±2.4)
BOEC-S	442	387 (87.6±1.2)	105 (24.1±2.0)	145 (33.5±2.8)	147 (34.3±3.8)
BOEC-E	424	379 (89.4±1.2)	92 (21.7±3.2)	132 (31.0±4.0)	151 (35.6±3.9)
BOEC-CM	510	447 (87.9±0.6)	141 (27.7±2.6)	182 (36.0±1.6)	192 (39.3±2.1)
BOEC-ECM	530	465 (87.6±1.5)	141 (26.8 ±2.2)	174 (33.3±2.5)	186 (35.8±2.7)

n: Total number of presumptive zygotes placed in culture.

**Fig 2:**
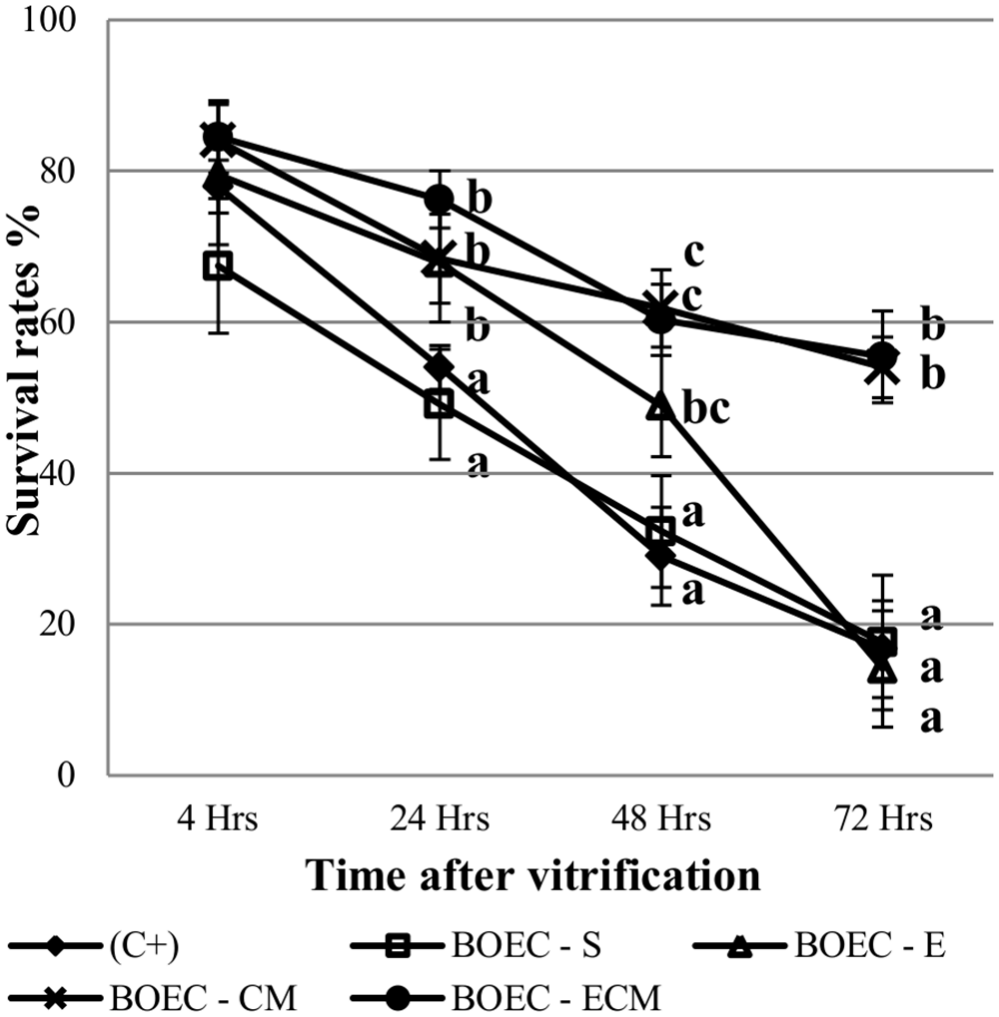
Survival rate after vitrification and warming of D7 blastocysts co-cultured with different types of BOEC (Suspension Cells: BOEC-S [n = 69], Extended Culture: BOEC-E [n = 53]) or Conditioned Media (from fresh: BOEC-CM [n = 70], or extended culture monolayers-BOEC-ECM [n = 66]) and Control+FCS (C+; n = 113). ^a,b,c^Different superscripts indicate significant differences at given time points (p<0.05).

In contrast, the survival rates of vitrified/warmed blastocysts produced in BOEC-E and CM was significantly higher when compared to BOEC-S and C^+^ groups at 24h (67.8%; 68.4%; 72.6% vs 49.1%; 54.0% respectively, p<0.05). At 72 h only blastocysts cultured in CM from BOEC-E survived significantly higher than BOEC-E, BOEC-S and C^+^ groups (54.0%; 55.4% vs 14.1%; 17.6%; 16.7% respectively, p<0.05) (Fig 2).

These differences in cryotolerance were also reflected in a significant higher number of TE cells in embryos cultured in BOEC-E, CM or ECM compared to BOEC-S and C^+^ groups (70.2; 72.1; 71.1 vs 68.7; 67.9 respectively, p<0.05). The total embryo cell number similar for all groups (range: 152.1 to 167.6) (Table 3).

**Table 3:** Effect of co-culture with different types of BOEC and Conditioned Media on blastocyst nuclei number.

	n	Total nuclei Mean ± S.E	ICM nuclei Mean ± S.E	ICM% ± S.E	TE nuclei Mean ± S.E	TE% ± S.E	Ratio ICM/TE
**Control (C**^**+**^**)**	44	152.1±4.7	47.7±1.7	32.1±1.2^a^	104.4±4.2	67.9±1.2^a^	0.5±0.02
**BOEC-S**	44	158.8±5.3	48.6±1.6	31.3±1.0^a^^c^	110.2±4.5	68.7±1.0^a^^c^	0.5±0.02
**BOEC-E**	44	161.9±4.1	47.5±1.8	29.8±1.2^a^^c^^d^	114.4±3.7	70.2±1.1^b^^c^	0.4±0.02
**BOEC-CM**	41	163.8±5.4	45.6±1.8	27.8±0.7^b^^d^	118.3±4.2	72.1±0.7^b^	0.4±0.01
**BOEC-ECM**	42	167.6±6.9	46.3±1.5	28.9±1.2^b^^c^	121.3±6.5	71.1±1.2^b^	0.4±0.02

n: Number of blastocysts processed.

^a,b,c^ Values in the same column with different superscripts differ significantly (p< 0.05).

### BOEC conditioned media contain extracellular vesicles

**Fig 3:**
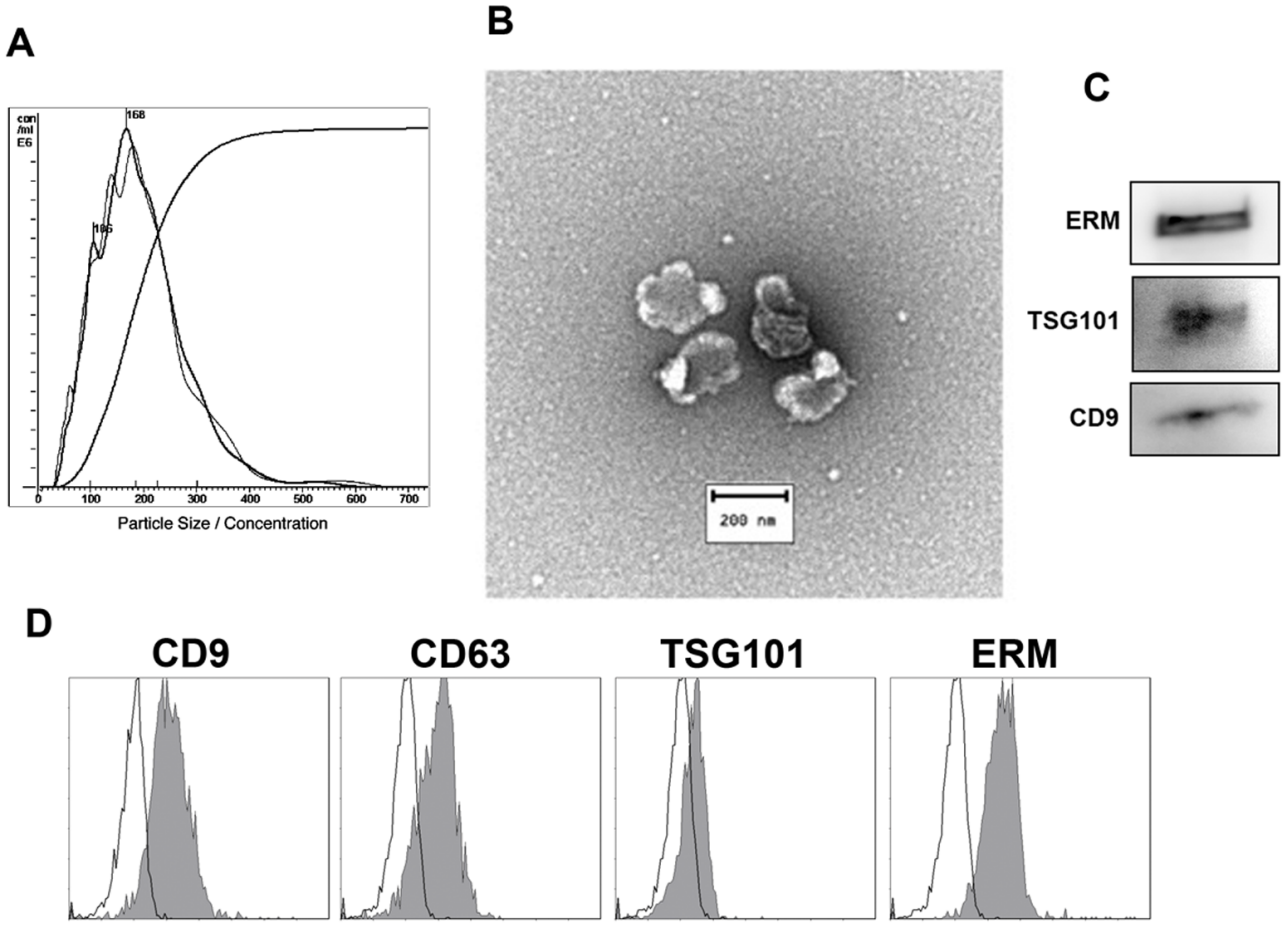
Characterization of vesicles isolated from BOEC-CM. **A**- Nanoparticle tracking analysis (NTA) of a representative EV sample. **B**- Transmission electron microscope image of negative-stained BOEC-EVs. **C-** Western-blot analysis of BOEC-EV lysates with EV markers. **D-** Bead-assisted flow cytometry analysis of EV isolated from BOEC-ECM. EV-coupled beads were stained for CD9, CD63, TSG101 and ERM EV markers. Negative control is depicted as an empty plot.

EVs were isolated from BOEC-CM by ultracentrifugation. Both nanoparticle tracking analysis (NTA) and transmission electron microscopy revealed that the isolates contained a relatively homogeneous population of vesicles of 150–200 nm in diameter (Fig 3A and 3B). Moreover, these vesicles expressed some of the classical markers described for exosomes (tetraspanins CD9 and CD63, TSG101 and ERM proteins) [47] (Fig 3C and 3D). NTA quantification of EVs revealed that the average concentration of secreted vesicles recovered from 10 mL of CM from a confluent BOEC monolayer in a 100 mm petri dish (≈5.5x10^6^ cells) was of 3x10^5^ EVs/mL. Therefore, and since 1:2 dilution of CM still retained the positive effect on *in vitro* embryo culture, we took 3x10^5^ EVs/mL as the starting concentration to use EVs in the following experiments.

### Extracellular vesicles secreted from BOEC have a positive effect on the quality of *in vitro* produced bovine embryos

Next, we wanted to analyze, whether BOEC-derived EVs were responsible for the positive effect of CM on the quality of bovine zygotes cultured *in vitro*. No differences were found in terms of cleavage rates (range: 86.2–89.8%) and blastocyst yield on Day 7 (range: 26.8–32.1), Day 8 (37.8–43.4) or Day 9 (range: 40.9–46.0) between C^+^ and the different groups supplemented with different concentrations of EVs, either fresh (F-EV) or frozen (Fr-EV), as shown in Table 4.

**Table 4:** Effect of culture with BOEC-EV at different concentrations on embryo development in vitro.

				Blastocyst yield		
	BOEC-EV Dilutions	n	Cleavage n (%±S.E)	Day 7 n (%±S.E)	Day 8 n (%±S.E)	Day 9 n (%±S.E)
**Control (C**^**+**^**)**		877	778 (88.8±1.0)	241 (27.5±1.2)	329 (37.8±1.7)	357 (4 0.9±1.8)
**F-EV**	**100%**	777	695 (89.6±1.1)	217 (28.3±1.2)	321 (41.2±2.2)	356 (4 5.5±2.3)
	**50%**	776	688 (88.6±1.1)	236 (31.2±2.4)	331 (43.4±3.1)	355 (46.0±2.9)
	**25%**	772	668 (86.2±1.2)	242 (32.1±2.2)	313 (41.2±2.8)	351 (46.0±2.7)
**Fr-EV**	**100%**	814	730 (89.9±0.9)	217 (26.8±1.0)	315 (38.7±2.2)	348 (42.7±1.8)
	**50%**	811	709 (87.2±0.7)	240 (30.2±1.9)	314 (39.7±2.8)	349 (44.1±3.0)
	**25%**	795	703 (88.6±0.9)	245 (30.9±1.7)	324 (40.5±2.0)	359 (45.1±2.0)

F-EV: Fresh EV. Fr-EV: Frozen/thawed EV. n: Total number of presumptive zygotes placed in culture.

**Fig 4:**
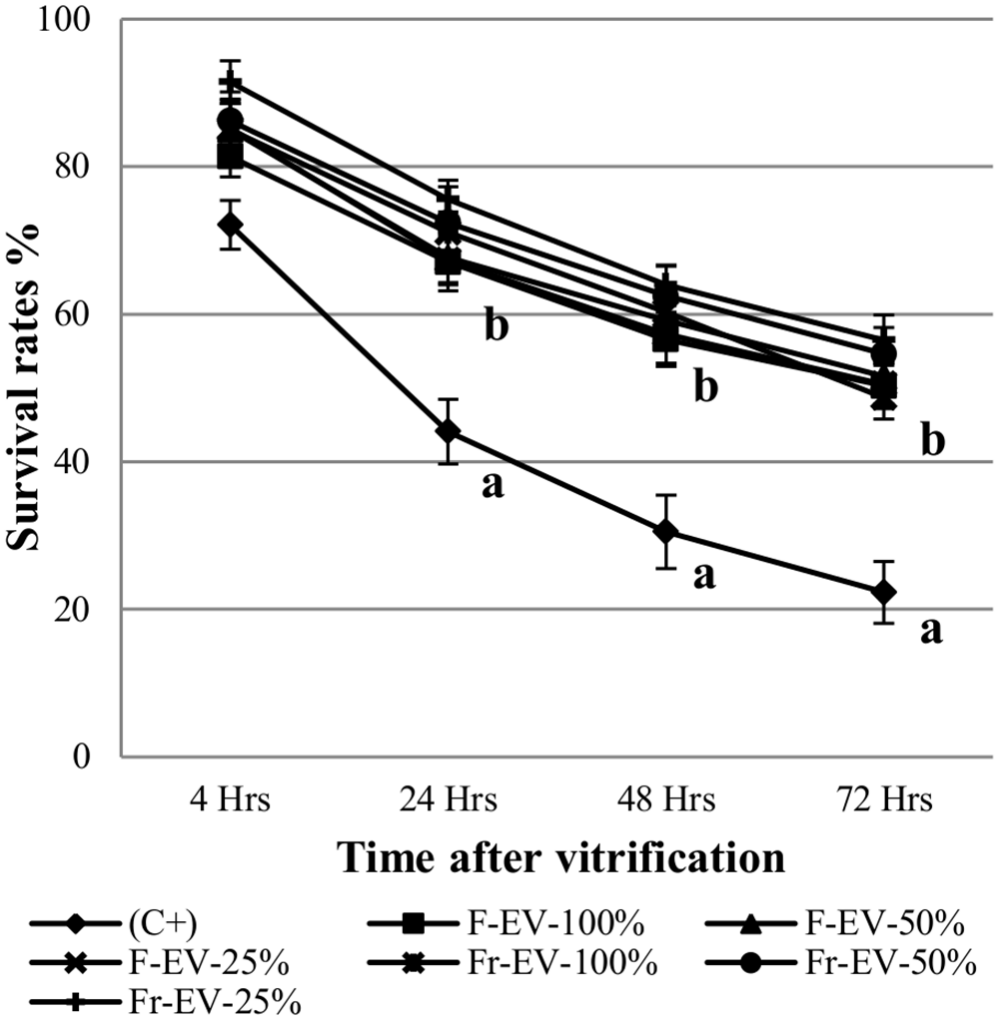
Survival rate after vitrification and warming of Day 7 blastocysts cultured with different concentrations (100, 50, 25%) of recently purified (F-EV; n = 96, 110, 94 respectively) or frozen/thawed (Fr-EV; n = 83, 86, 87 respectively)) BOEC extracellular vesicles. ^a,b^Different superscripts indicate significant differences at given time points (p<0.05).

Interestingly, embryos cultured with EVs, irrespective of concentration and processing, survived significantly higher than C^+^ group at all-time points (range at 72h: 48.7–56.5% vs 22.3% respectively, p<0.05) (Fig 4).

Similarly, blastocysts cultured in the presence of BOEC EVs had significantly more cells than the C^+^ group (range: 177.1–191.1 vs 160.4 respectively, p<0.05) and also more TE cells (range: 127.5–131.8 vs 111.5 respectively, p<0.05), as shown in Table 5.

**Table 5:** Effect of culture with BOEC-EV at different concentrations on blastocyst nuclei number.

	BOEC-EV Dilutions	n	Total nuclei Mean ± S.E	ICM Nuclei Mean ± S.E	ICM % ± S.E	TE nuclei Mean ± S.E	TE % ± S.E	Ratio ICM/TE
**Control (C**^**+**^**)**		40	160.4±7.3^a^	48.9±3.6	30.3±1.2	111.5±5.4^a^	69.7±1.2	0.5±0.02
**F-EV**	**100%**	40	180.7±8.2^b^	51.6±2.8	28.8±1.1	129.1±6.3^b^	71.2±1.1	0.4±0.02
	**50%**	41	175.3±8.0^a^^b^	47.9±2.8	27.1±0.8	127.5±5.8^b^	72.9±0.8	0.4±0.02
	**25%**	41	182.5±7.5^b^	51.6±3.0	28.1±1.0	130.8±5.4^b^	71.9±1.0	0.4±0.02
**Fr-EV**	**100%**	40	177.1±7.1^b^	49.0±2.9	27.3±0.9	128.0±5.0^b^	70.7±0.9	0.4±0.02
	**50%**	39	184.1±9.0^b^	56.2±3.5	30.6±1.3	127.9±6.5^b^	69.4±1.3	0.5±0.03
	**25%**	38	191.1±8.6^b^	59.4±3.7	30.4±1.1	131.8±5.6^b^	69.6±1.1	0.5±0.02

n: Number of blastocysts processed. F-EV: Fresh EV. Fr-EV: Frozen/thawed EV.

^a,b^Values in the same column with different superscripts differ significantly (p< 0.05).

When we assessed whether the media employed for the culture of BOEC previous to the isolation of EVs had an effect on embryo development, again no differences were found in cleavage rates between groups (range: 82.6–86.8%). However, blastocyst yield of the C^+^ group was significantly higher than C^-^, and both EVs (DMEM and TCM199) groups, on Days 7, 8 and 9 (Day 9: 29.9% vs 23.6%; 24.5%; 23.3% respectively, p<0.05) as shown in Table 6.

**Table 6:** Effect of culture with EV secreted by BOEC cultured in DMEM and TCM199 on embryo development in vitro.

				Blastocyst yield		
	BOEC-EV from	n	Cleavage n (%±S.E)	Day 7 n (%±S.E)	Day 8 n (%±S.E)	Day 9 n (%±S.E)
**Control (C**^**+**^**)**		490	405 (82.6±1.0)	117 (24.2±1.2) ^a^	134 (27.4±1.0) ^a^	146 (29.9±1.8) ^a^
**Control (C**^**-**^**)**		590	504 (85.5±1.1)	77 (13.7±1.5) ^b^	122 (20.8±0.6) ^b^	138 (23.6±0.8) ^b^
**Fr-EV**	**DMEM**	631	541 (85.1±1.3)	94 (16.1±1.9) ^b^	132 (22.6±2.6) ^b^	144 (24.5±2.3) ^b^
	**TCM199**	630	552 (86.8±1.5)	76 (12.3±1.3) ^b^	122 (20.3±1.9) ^b^	142 (23.3±1.5) ^b^

n: Total number of presumptive zygotes placed in culture.

^a,b^Values in the same column with different superscripts differ significantly (p< 0.05).

**Fig 5:**
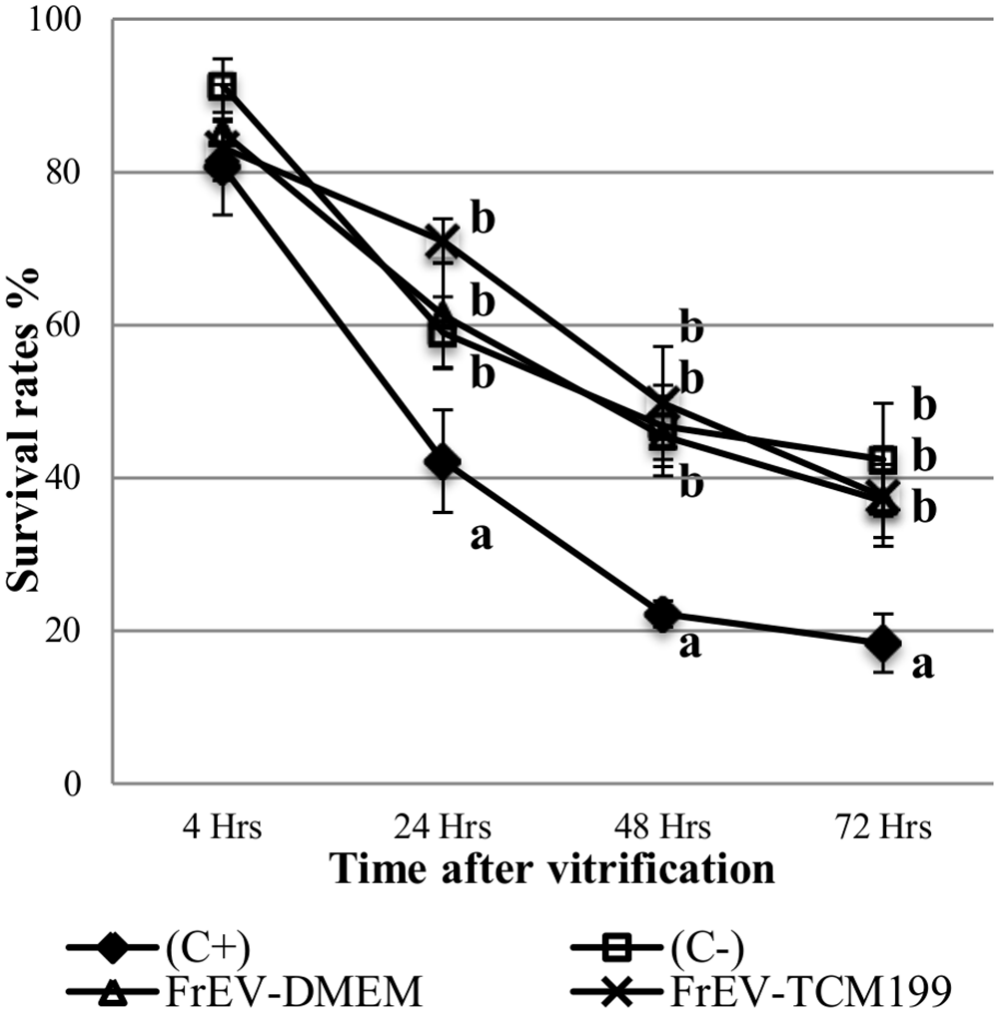
Survival rate after vitrification and warming of D7-8 blastocysts cultured with extracellular vesicles (EV) secreted by BOEC cultured in DMEM (n = 61), TCM199 (n = 59) or in Control+FCS (C+; n = 46) and C-: Control-FCS (C+; n = 50). ^a,b,^Different superscripts indicate significant differences at given time points (p<0.05).

The survival rate after vitrification and warming of embryos cultured with EVs (DMEM or TCM199) and without FCS was significantly higher than the C+ group at all-time points (72 h: 37.0%; 36.7% vs 18.4% respectively, p<0.05). However, no differences were found between C- and EVs groups (Fig 5).

In terms of cell number, blastocysts produced with EVs (DMEM and TCM199) and without FCS and C- had lower number of total (range: 133.6–142.7) and TE cells (range: 101.4–108.6) compared to C+ (155.9 and 117.6 respectively) (Table 7).

**Table 7:** Effect of culture with EV secreted by BOEC cultured in DMEM and TCM199 on blastocyst nuclei number.

	BOEC-EV from	n	Total nuclei Mean ± S.E	ICM Nuclei Mean ± S.E	ICM% ± S.E	TE nuclei Mean ± S.E	TE % ± S.E	Ratio ICM/TE
**Control (C**^**+**^**)**		32	155.9±10.8	38.2±2.6	25.4±1.1	117.6±9.1	74.5±1.1	0.3±0.02
**Control (C**^**-**^**)**		31	133.6±7.7	31.2±2.2	23.9±1.4	101.4±6.5	76.0±1.4	0.3±0.03
**Fr-EV**	**DMEM**	31	142.7±8.7	34.0±1.8	24.6±1.0	108.6±7.4	75.3±1.0	0.3±0.01
	**TCM199**	31	141.7±7.6	35.2±2.4	24.8±1.2	106.4±5.4	75.0±1.2	0.3±0.02

n: Number of blastocysts processed.

**Fig 6:**
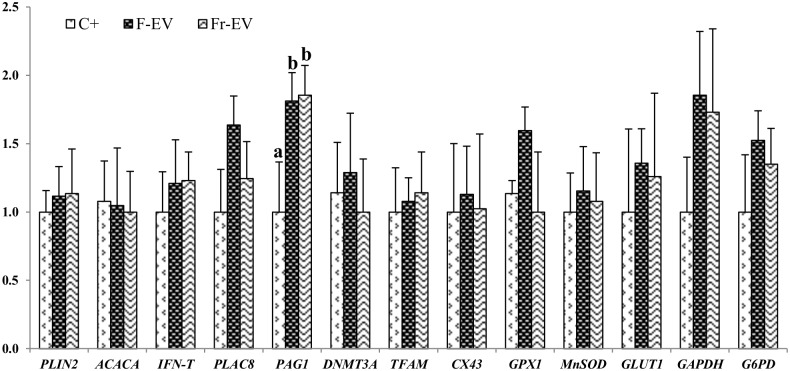
Relative mRNA transcription in bovine in vitro blastocysts (D7 p.i) cultured with or without (C+) EV secreted by BOEC fresh (F-EV) and frozen (Fr-EV) of genes related with fatty acid metabolism (PLIN2, ACACA), implantation (IFN-T, PLAC8, PAG1), epigenetics (DNMT3A, TFAM), gap junctions (CX43) and oxidative stress (GPX1,MNSOD, GLUT1, GAPDH, G6PD). Data are relative to mean of internal genes H2AFZ, and 18S. Data presented are mean±SE. ^a.b^ Different superscripts indicate significant differences for each gene (p<0.05).

**Fig 7:**
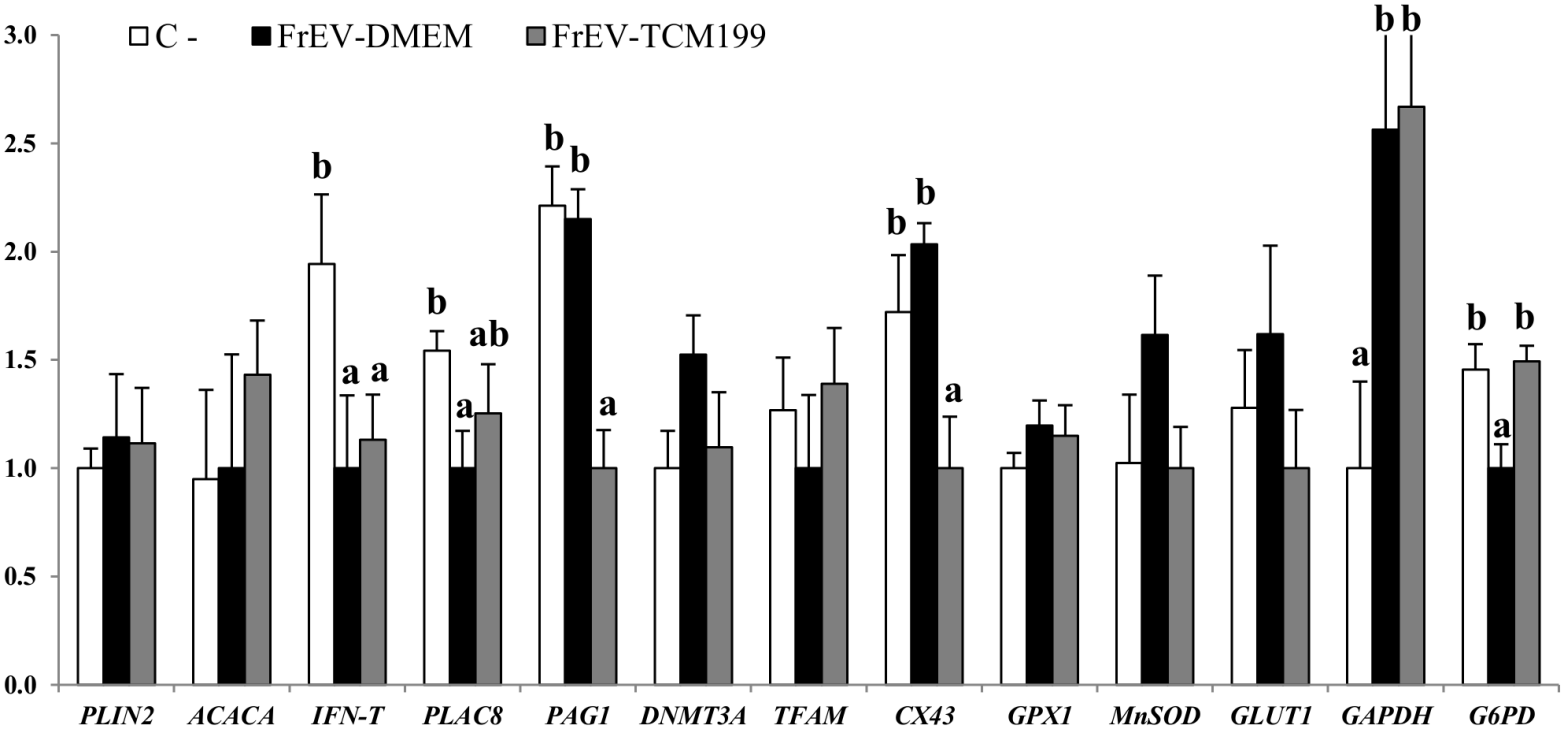
Relative mRNA transcription in bovine in vitro blastocysts (D7-8 p.i) cultured with or without (C-) EV secreted by BOEC cultured in DMEM and TCM199 of genes related with fatty acid metabolism (*PLIN2*, *ACACA*), implantation (*IFN-T*, *PLAC8*, *PAG1*), epigenetics (*DNMT3A*, *TFAM*), gap junctions (*CX43*) and oxidative stress (*GPX1*,*MNSOD*, *GLUT1*, *GAPDH*, *G6PD*). Data are relative to mean of internal genes H2AFZ, and 18S. Data presented are mean±SE. ^a.b^ Different superscripts indicate significant differences for each gene (p<0.05).

We next analyzed the expression levels of different genes, including housekeeping genes (histone *H2AFZ*, 18 s ribosomal RNA (*18S*)); fatty acid related genes such as periplin 2 (*PLIN2*), and acetyl-Coa carboxylase alpha (*ACACA*); implantation-related genes (interferon tau (*IFN-t*), placenta specific 8 (*PLAC8*) and pregnancy associated glycoprotein 1 (*PAG1*)); epigenetics-related genes (DNA methyltransferase 3A (*DNMT3A*), transcription factor A, mitochondrial (*TFAM*), gap junctions gene connexin 43 (*Cx43*)) and genes involved in the regulation of oxidative stress such as gluthathione peroxidase 1 (G*PX1*), manganese superoxide dismutase (*MnSOD*), solute carrier family 2 (facilitated glucose transporter), member 1 (*SCL2A1*, previously known as *GLUT1*), glyceraldehyde-3-phosphate dehydrogenase (*GAPDH*) and glucose 6 phospathe dehydrogenase (*G6PD*). The expression level of *PAG1*, an implantation related gene, was upregulated in blastocysts cultured in the presence of FCS supplemented with fresh (F-EV) or Frozen (Fr-EV) extracellular vesicles when compared with C^+^. No differences were observed for the rest of transcripts studied (Fig 6). In contrast, in the absence of FCS, the expression level of *IFN-τ* was downregulated in both EV groups (FrEV-DMEM and FrEV-TCM199) compared to C^-^. *PLAC8* was downregulated in FrEV-DMEM while *PAG1* and *Cx43* were downregulated in FrEV-TCM199 group. *GAPDH* was upregulated in both EVs groups; and *G6PD* was downregulated in the FrEV-DMEM group (Fig 7).

### Depletion of extracellular vesicles from fetal calf serum improves the quality of bovine embryos produced *in vitro*

**Fig 8:**
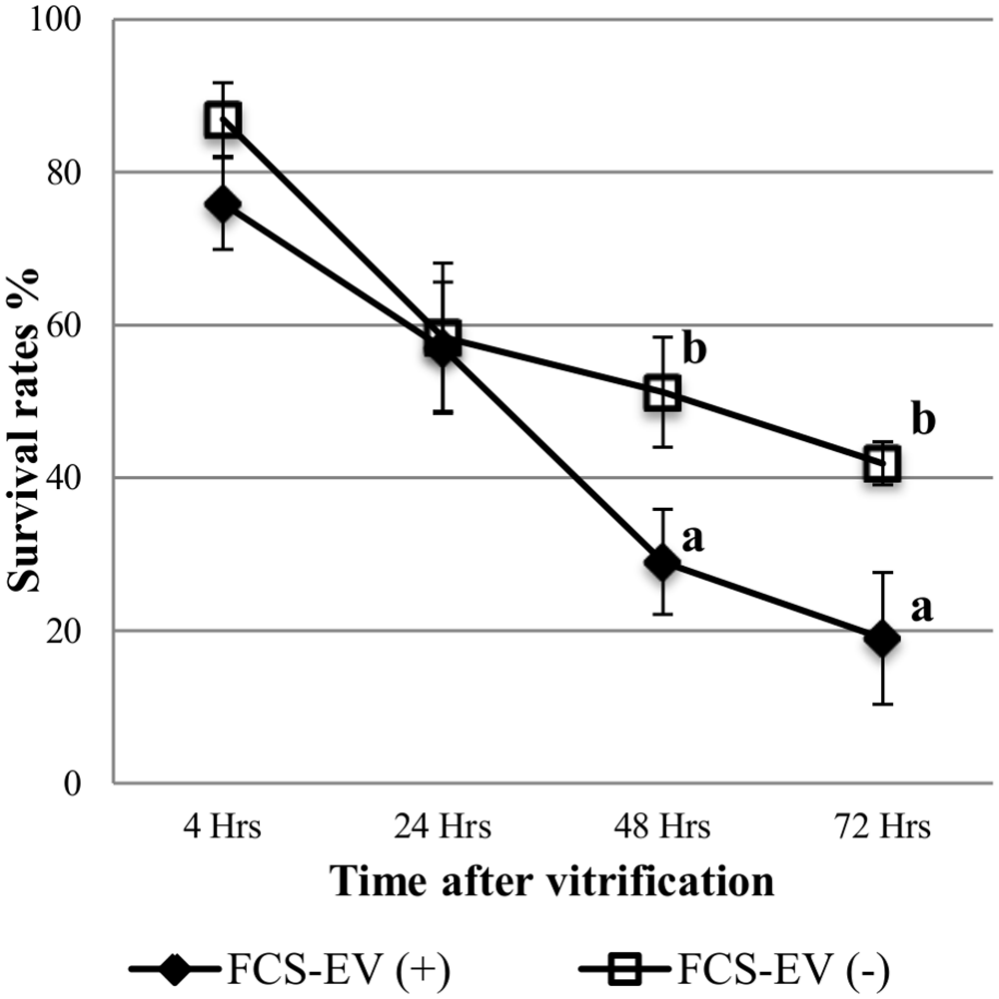
Survival rate after vitrification and warming of D7 blastocysts cultured with normal FCS (containing EV; n = 46) or EV-depleted FCS; n = 49. ^a,b^Different superscripts indicate significant differences at given time points (p<0.05).

Since FCS is also a source of EVs, we wanted to analyze the effect of those EVs present in FCS on the *in vitro* bovine embryo development and quality. No differences were found between FCS-EV(+) and FCS-EV(-) for cleavage rate (84.0±3.3% vs.82.0±0.7%) or blastocyst yield on Day 7 (22.7±2.3%vs.28.8±5.6%) or d9 (34.0±3.4vs.38.1±5.4%) (Table 8). However, after vitrification/warming, significantly more embryos survived at 48 and 72 h in FCS-EV(-) compared with FCS-EV(+) group (51.2±8.2% and 41.9±2.8% vs. 29.2±6.9% and 19.0±8.6%, respectively) (p<0.05) (Fig 8).

**Table 8:** Effect of culture in presence (+) or absence (-) of FCS-EV on embryo development *in vitro*.

			Blastocyst yield		
	n	Cleavage n (% ± S.E)	Day 7 n (% ± S.E)	Day 8 n (% ± S.E)	Day 9 n (% ± S.E)
**FCS-EV (+)**	333	281 (84.0±3.3)	78 (22.7±2.3)	106 (31.4±4.0)	115 (34.0±3.4)
**FCS-EV (-)**	321	264 (82.0±0.7)	95 (28.8±5.6)	120 (35.8±4.2)	130 (38.1±5.4)

n: Total number of presumptive zygotes placed in culture.

## Discussion

In this study, we report a highly standardizable method to improve the quality of the produced bovine embryos in *in vitro* culture. Firstly, we report a methodology to established a BOEC line that can be used successfully after freezing and thawing thus avoiding the lack of reproducibility between replicates with different primary cultures. Secondly, we provide firm evidence that fresh or frozen BOEC CM improve blastocyst quality to the same extent as classical co-culture with fresh BOEC monolayers. Finally, we can reproduce this improving effect by the sole addition of EVs isolated from the conditioned media of this established cell line.

It is well known that culture environment during embryo development has an impact on the quality of the produced embryos in terms of cryotolerance [1,14], ultrastructure morphology [48]; embryo cell number [49] and gene expression [50,51]. Ellington *et al*. reported superior development of bovine zygotes and early embryos in simple medium with BOEC monolayers, compared with a complex medium [52]. Recently, a study by Cordova *et al*. confirmed that the presence of BOEC at the early stages of embryo development, up to four days, improves embryo development and embryo quality in terms of specific gene transcripts, concluding that this period reflects the *in vivo* conditions where the embryo is still in the oviduct [20]. We clearly showed here that an extended culture BOEC monolayer can be used successfully for co-culture with no differences in embryo development (Day 7–9: ≈35%) when compared either with co-culture with fresh recovered cells or normal culture in SOF. This factor gives a great advantage over the classical co-culture systems since it helps to provide homogenous results.

However, the main aim of embryo co-culture is to take advantage of oviductal embryotrophic substances such as growth factors [53,54]. Thus, the use of CM for *in vitro* culture would avoid undesired confounding effects of the presence of co-cultured cells/tissue [55]. In addition, CM can be prepared in large quantities, frozen and used when needed [56]. Our results support the idea that CM from the extended culture BOEC monolayer had a similar impact on blastocyst quality than co-culture with fresh BOEC, further reinforcing the advantages of using an established BOEC line, in agreement with Mermillod *et al*. who demonstrate that BOEC CM in absence of FCS induce differential effects of embryonic development in terms of cleavage and blastocyst rates [56].

The positive effect of BOEC CM could be due to soluble factors or to the presence in this medium of EVs secreted by these cells. Electron microscopy and nanoparticle tracking analysis support the conclusion that EVs are present in these conditioned media from the extended culture BOEC monolayer. By classical ultracentrifugation methods [45] we obtained 3x10^5^ EVs/ml from initial 10 ml of BOEC CM as assessed by Nanosight. To date only Burns *et al*. have demonstrated the presence of EVs in the reproductive tract of ruminants [57]. These authors isolated exosomes (30 to 100 nm in diameter) and microvesicles from uterine luminal fluid of pregnant and cyclic ewes, containing specific proteins, miRNAs, and mRNAs. These studies provide evidence for pre-implantation communication of the conceptus and endometrium via cell secreted or shed vesicles. Recently, Al-Dossary *et al*. showed in mice the effect of oviductal exosomes on sperm motility and fertility [58]. However, to our knowledge this is the first report where EVs from BOEC have been isolated, morphologically characterized and used in *in vitro* embryo culture.

The addition of BOEC EVs at different concentrations to embryo culture in the presence of FCS and in a serum-free media, produced a positive effect on embryo cryotolerance with a significantly higher survival rate after vitrification and warming, overcoming the negative effect of serum. Moreover, the percentage of TE cells in embryos cultured with CM was significantly higher than the BOEC co-culture and control groups. TE cells are crucial for blastocoele re-expansion and maintenance after cryopreservation. Thus, higher lipid contents in TE cells [59] render them particularly susceptible to damage during cryopreservation. Moreover, in cattle, trophoblastic cells play a crucial role around Day 14 when intense trophoblastic proliferation begins together with increased trophoblastic secretion of the pregnancy recognition factor interferon-tau (IFNT) [60]. IFNT regulates the expression of various uterine-derived factors responsible for placental attachment, modify the uterine immune system, and regulate early conceptus development [61]. Therefore, trophoblastic cells have an essential role in implantation and placentation.

The addition of foetal bovine serum to the culture media accelerates embryonic developmental kinetics and increases the number of embryonic cells [62]. However, embryos cultured in the presence of serum have a lower level of compaction at the morula stage [63], exhibit a greater accumulation of lipid droplets in the cytoplasm [13], have lower cryotolerance [13] and exhibit alterations in gene expression [64] compared to *in vivo* produced embryos. In addition, serum has been linked to the "Large Offspring Syndrome" (LOS) [65], that causes the birth of large calves with musculoskeletal disorders, alterations in the development of the allantois and defects in vascularization and development of the placenta, showing a smaller area of maternal-fetal contact [65,66]. Therefore, the need to develop a serum-free *in vitro* culture system/media is evident.

Serum also contains EVs with unknown function on embryo development and quality. Our data suggest that EVs from FCS have a deleterious effect on embryo quality. Thus, EVs from FCS may be at least partially responsible for its consequences in short and long-term embryo/foetal development. Importantly, our date also suggest that addition of BOEC EVs can compensate for the deleterious effect of FCS EVs. Hence, our results support the hypothesis that EVs from BOEC, but not those found in FCS, have a positive effect on the quality of *in vitro* produced bovine embryos, suggesting that EVs may have a determinant function in the communication between the oviduct and the embryo in the early stages of development.

Gene expression analysis of blastocysts cultured with BOEC EVs in the presence of serum did not show differences compared to the control group, apart from *PAG1*, an implantation related gene, member of aspartic proteinase gene family, considered a product of binucleated cells in ruminants trophectoderm [27,67,68], which was highly expressed in BOEC EVs groups. In cows, pregnancy-associated glycoproteins are released into the maternal circulation soon after implantation (i.e. around Day 25) and thereafter, concentrations rise until parturition. Plasma *PAG1* levels have been used for pregnancy diagnosis and as a marker of placental/foetal connection [69,70]. High expression levels of *PAG1* may be associated to late gestation, while with lower expression, would correspond to Mid-gestation considering the classification of [71].

The fact that no clear differences were observed in gene expression, in clear contrast with the significant effect on cryotolerance and embryo cell number, could be partially explained by the masking effect of the presence of serum. In this regard, in the absence of serum, *IFN-τ* and *PLAC8* expression levels were down regulated in EVs groups compared to C^-^, indicating a better quality embryo. A bovine embryo begins to express IFN-τ at the blastocyst stage [72] and expression is primarily dependent on the presence of a functional TE [73,74]. Kubisch *et al*. reported a negative relationship between early *IFN-τ* production and developmental competence [75], which was later confirmed by comparing *in vivo*- and *in vitro*-produced blastocysts, showing that an early and high expression of *IFN-τ* indicates poor quality embryo [76]. Although PLAC8 was reported to be a gene related with successful fetal development, playing an important role in placental development and feto-maternal interaction [77], and associated with live birth of *in vitro* produced embryos [78]; *in vivo* produced embryos down-regulate *PLAC8* compared to *in vitro* counterparts [79].

Additional markers of improved embryo quality would correspond to the upregulated expression of *CX43* and *GAPDH*. *CX43* is related with compaction and cell to cell adhesion [64,76], and high expression of *CX43* has been associated with better quality embryos and increased cryotolerance [80]. With regard to GAPDH, it has multiple functions independent of its role in energy metabolism. Increased GAPDH gene expression and enzymatic function is associated with cell proliferation [81]. In many studies *GAPDH* has been used as a housekeeping gene [82]. However, Garcia-Herreros *et al*. found higher level of GAPDH protein in faster developing male embryos compared to female [83].

Our results also suggest that the media (DMEM or TCM-199) used to culture BOEC did not affect the capacity of EVs to improve embryo development and quality, although some specific effects were observed on embryo gene expression.

The G6PD gene, an indicator of the pentose phosphate pathway activity [84], was either up-or down-regulated in embryos cultured with EVs depending on the media used for BOEC culture. A lower expression of *G6PD* has been observed in *in vitro* produced bovine embryos [85] and has been related with lower quality. However, in other studies a significantly higher *G6PD* expression has been observed in *in vitro*-produced embryos compared with *in vivo* cultured in the ewe oviduct [50] or obtained *in vivo* [86,87]. Also, the expression of this gene can be influenced *in vitro* by other factors such as sex of embryo, origin of embryo or respiration rate [85,88].

In conclusion, by trying to mimic the intercellular communications between oviductal tissue and embryo, we provide evidence that EVs isolated from the conditioned medium of an extended culture BOEC monolayer improve embryo quality and induce cryoprotection in *in vitro* cultures. This is the first study in which EVs from BOEC have been isolated, morphologically characterized and successfully used in *in vitro* embryo culture as an alternative to serum to improve the quality of the produced embryos. Future studies on EV proteome and transcriptome will further identify the molecular mechanisms behind this maternal-embryo communication that affects the embryo development *in vitro*.

## Supporting Information

S1 Fig Immunofluorescence analysis of the pattern of expression of cadherin, cytokeratins and vimentin in confluent monolayers of BOEC-E cultures. (TIF)
