# Supplementary figures and images for: Extracellular Vesicles from BOEC in In Vitro Embryo Development and Quality
Source: PLoS One. 2016 Feb 4;11(2):e0148083. doi: 10.1371/journal.pone.0148083 (PMC4742056; doi:10.1371/journal.pone.0148083)

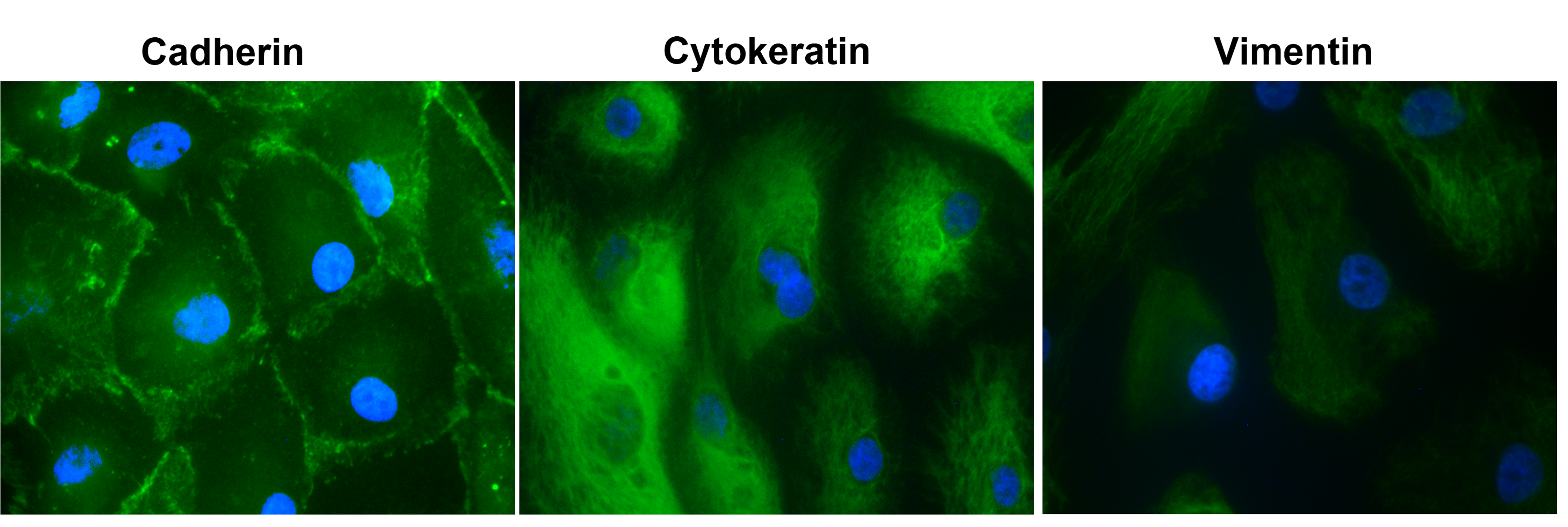

Supplement: S1 Fig — (TIF) [file pone.0148083.s001.tif]
